# Supplementary material for: A Fine-Scale Hotspot at the Edge: Epigean Arthropods from the Atacama Coast (Paposo-Taltal, Antofagasta Region, Chile)
Source: Insects. 2021 Oct 8;12(10):916. doi: 10.3390/insects12100916 (PMC8540830; doi:10.3390/insects12100916)
Supplement: Supplementary file 1 [file insects-12-00916-s001.zip › insects-1357057-supplementary.pdf]

Table S1 Taxonomic references for specific groups

Pizarro-Araya et al. 2021. A Fine-Scale Hotspot at the Edge: Epigean Arthropods from the Atacama Coast (Paposo-Taltal, Antofagasta Region, Chile).

- Arachnida, Araneae: Platnick and Shadab (1982), Coyle (1986), Goloboff (1995), Huber (2000), Ramírez (2003), Platnick et al. (2005), Laborda et al. (2013), Brescovit and Sánchez-Ruiz (2016) and Magalhaes et al. (2017);

- Solifugae: Kraus (1966), Muma (1971), and Maury (1987);

- Scorpiones: Ojanguren-Affilastro (2002, 2005), Ojanguren-Affilastro and Ramírez (2009), Ojanguren-Affilastro and Kovarík (2013), Ojanguren-Affilastro and Pizarro-Araya (2014) and Ojanguren-Affilastro et al. (2016, 2018);

- Insecta: Kulzer (1955, 1958, 1959), Peña (1966, 1971, 1973, 1974, 1980, 1994, 1995), Dajoz (1967), Kaszab (1969), Marcuzzi (1976), Artigas (1994), Moore (1985, 1994, 2017), Estrada and Solervicens (1999), Roig-Juñent and Domínguez (2001), Pizarro-Araya and Jerez (2004), Ferrú and Elgueta (2011), Flores and Pizarro-Araya (2012, 2014, *in rev*) and Mondaca et al. (2019).

References

Artigas, J.N., 1994. Entomología Económica. Insectos de Interés Agrícola, Forestal, Médico y Veterinario (Nativos, Introducidos y Susceptibles de ser Introducidos). Volúmenes I & II Ediciones Universidad de Concepción, Concepción, Chile.

Brescovit, A.D., Sánchez-Ruiz, A., 2016. Descriptions of two new genera of the spider family Caponiidae (Arachnida, Araneae) and an update of *Tisentnops* and *Taintnops* from Brazil and Chile. Zookeys 622, 47–84. https://doi.org/10.3897/zookeys.622.8682

Coyle, F.A., 1986. *Chilehexops*, a new funnelweb mygalomorph spider genus from Chile (Araneae, Dipluridae). Am. Mus. Novit. 2860, 1–10.

Dajoz, R., 1967. Contribution a l'etude des Coleopteres Lathridiidae du Chili. Biologie de l'Amerique Australe 3, 587–609.

Estrada, P., Solervicens, J., 1999. Revisión taxonómica de las especies chilenas del género *Arthrobrachus* Solier, 1849 (Coleoptera: Melyridae). Acta Entomológica Chil. 23, 41–81

Ferrú, M.A., Elgueta, M., 2011. Lista de coleópteros (Insecta: Coleoptera) de las regiones de Arica y Parinacota y de Tarapacá, Chile. Bol. Mus. Nac. Hist. Nat. 60, 9–61.

Flores, G.E., Pizarro-Araya, J., 2012. Systematic revision of the South American genus *Praocis* Eschscholtz, 1829 (Coleoptera: Tenebrionidae). Part 1: Introduction and subgenus *Praocis* s. str. Zootaxa 3336, 1–35. https://doi.org/10.11646/zootaxa.3336.1.1

Flores, G.E., Pizarro-Araya, J., 2014. Towards a revision of the South American genus *Praocis* Eschscholtz (Coleoptera: Tenebrionidae), with estimation of the diversity of each subgenus. ZooKeys 415, 53–80. https://doi.org/10.3897/zookeys.415.6656

Flores, G.E., Pizarro-Araya, J., *In Rev*. Morphology of sensilla on foretibia, fossorial adaptations and revision of the subgenus *Praocis* (*Mesopraocis*) (Coleoptera: Tenebrionidae: Pimeliinae). An. Acad. Bras. Cienc.

Goloboff, P.A., 1995. A revision of the South American spiders of the family Nemesiidae (Araneae, Mygalomorphae). Part I: species from Perú, Chile, Argentina, and Uruguay. Bull. Am. Mus. Nat. Hist. 224, 1–189.

Huber, B.A., 2000. New world pholcid spiders (Araneae: Pholcidae): A revision at generic level. Bull. Am. Mus. Nat. Hist. 254, 4–348.

Kaszab, Z., 1969. The scientific results of the Hungarian soil Zoological Expeditions to South America. 17. Tenebrioniden aus Chile (Coleoptera). Opusc. Zool. Budapest 9, 291–337.

Kraus, O., 1966. Solifugen aus Chile (Arachnida). Senckenb. Biol. 47, 181–184.

Kulzer, H., 1955. Monographie der Scotobiini (Zehnter Beitrag zur Kenntnis der Tenebrioniden). Entomol. Arb. Mus. G. Frey Tutzing bei Münch. 6, 383–478.

Kulzer, H., 1958. Monographie der südamerikanischen Tribus Praocini (Col.) (16 Beitrag zur Kenntnis der Tenebrioniden). Entomol. Arb. Mus. G. Frey Tutzing bei Münch. 9, 1–105.

Kulzer, H., 1959. Neue Tenebrioniden aus Südamerika (Col). (18. Beitrag zur Kenntnis der Tenebrioniden). 3. Verschiedene Neue Arten. Entomol. Arb. Mus. G. Frey Tutzing bei Münch. 10, 523–567.

Laborda, A., Ramírez, M.J., Pizarro-Araya, J., 2013. New species of the spider genera *Aysenia* and *Aysenoides* from Chile and Argentina: description and phylogenetic relationships (Araneae: Anyphaenidae, Amaurobioidinae). Zootaxa 3731, 133–152. https://doi.org/10.11646/zootaxa.3731.1.6

Magalhaes, I.L.F., Brescovit, A.D., Santos, A.J., 2017. Phylogeny of Sicariidae spiders (Araneae: Haplogynae), with a monograph on Neotropical Sicarius. Zool. J. Linnean Soc. 179, 767–864. 10.1111/zoj.12442

Marcuzzi, G., 1976. New species of Neotropical Tenebrionidae (Coleoptera). Ann. Hist. Nat. Mus. Nat. Hung. 68, 117–139.

Maury, E.A., 1987. Consideraciones sobre algunos solífugos de Chile (Solifugae: Ammotrechidae, Daesiidae). Rev. Soc. Entomol. Arg. 44, 419–432.

Mondaca, J., Pizarro-Araya, J., Alfaro, F.M., 2019. Revision of the genus *Luispenaia* Martínez (Coleoptera: Scarabaeidae: Melolonthinae: Tanyproctini), with description of three new species from the Atacama Desert, Chile. Zootaxa 4615, 549–562. https://doi.org/10.11646/zootaxa.4615.3.8

Moore, T., 1985. Aporte al conocimiento de los Bupréstidos en Chile (Coleoptera: Buprestidae) Segunda nota. Rev. Chil. Entomol. 12, 113–139.

Moore, T., 1994. Revisión del género *Ectinogonia* Spinola para Chile (Coleoptera: Buprestidae). Bol. Soc. Biol. Concepc. 65, 153–166.

Moore, T., 2017. *Ectinogonia barrigai* nov. sp.: primera especie de bupréstido del Monumento Natural Paposo Norte, Región de Antofagasta, Chile (Coleoptera: Buprestidae). Rev. Chil. Entomol. 42, 5–10.

Muma, M.H., 1971. The Solpugids (Arachnida, Solpugida) of Chile, with descriptions of a new family, new genera, and new species. Am. Mus. Novit. 2476, 1–23.

Ojanguren-Affilastro, A.A., 2002. Nuevos aportes al conocimiento del género *Brachistosternus* en Chile, con la descripción de dos nuevas especies (Scorpiones, Bothriuridae). Bol. Soc. Biol. Concepc. 73, 37–46.

Ojanguren-Affilastro, A.A., 2005. Notes on the genus *Brachistosternus* (Scorpiones, Bothriuridae) in Chile, with the description of two new species. J. Arachnol. 33, 175–192. https://doi.org/10.1636/h03-20

Ojanguren-Affilastro, A.A., Ramirez, M.J., 2009. Phylogenetic analysis of the scorpion genus *Brachistosternus* (Arachnida, Scorpiones, Bothriuridae). Zool. Scr. 38, 183–198. https://doi.org/10.1111/j.1463-6409.2008.00367.x

Ojanguren-Affilastro, A.A., Kovarík, F., 2013. Bothriuridae. Illustrated catalog of scorpions. Part II. Bothriuridae; Chaerilidae; Buthidae I., genera *Compsobuthus, Hottentotta, Isometrus, Lychas,* and *Sassanidotus*, in: Kovarík, F., Ojanguren-Affilastro, A.A. (Eds.), Jakub Rolčík Publisher, Czech Republic, pp. 6–130.

Ojanguren-Affilastro, A.A., Pizarro-Araya, J., 2014. Two new scorpion species from Paposo, in the Coastal desert of Taltal, Chile (Scorpiones, Bothriuridae, *Brachistosternus*). Zootaxa 3785, 400–418. http://dx.doi.org/10.11646/zootaxa.3785.3.4

Ojanguren-Affilastro, A.A., Mattoni, C.I., Ochoa, J.A., Ramírez, M.J., Ceccarelli, F.S., Prendini, L., 2016. Phylogeny, species delimitation and convergence in the South American bothriurid scorpion genus *Brachistosternus* Pocock 1893: Integrating morphology, nuclear and mitochondrial DNA. Mol. Phylogenet. Evol. 94, 159–170. https://doi.org/10.1016/j.ympev.2015.08.007

Ojanguren-Affilastro, A.A., Pizarro-Araya, J., Ochoa-Cámara, J.A. 2018. Five new scorpion species of genus *Brachistosternus* (Scorpiones: Bothriuridae) from the deserts of Chile and Peru, with comments about some poorly studied diagnostic characters of the genus. Zootaxa 4531, 151–194. https://doi.org/10.11646/zootaxa.4531.2.1

Peña, L.E., 1966. Catálogo de los Tenebrionidae (Coleoptera) de Chile. Entomol. Arb. Mus. G. Frey Tutzing bei Münch. 17, 397‒453.

Peña, L.E., 1971. Revisión del género *Nycterinus* Eschscholtz, 1829 (Coleoptera-Tenebrionidae). Bol. Mus. Nac. Hist. Nat. 32, 129‒158.

Peña, L.E., 1973. Nuevas especies del género *Psammetichus* Latr. (Coleoptera-Tenebrionidae) para Chile y Perú. Rev. Chil. Entomol. **7**, 137‒144.

Peña, L.E., 1974. Los tenebriónidos del género *Thinobatis* Esch. (Coleoptera: Tenebrionidae). Bol. Mus. Nac. Hist. Nat. XLVIII, 243‒252.

Peña, L.E., 1980. Aporte al conocimiento de los tenebriónidos de América del Sur (Coleoptera: Tenebrionidae). Rev. Chil. Entomol. 10, 37‒59.

Peña, L.E., 1994. Nuevas especies de Tenebrionidae (Insecta-Coleoptera) de la Región Neotropical. Gayana 58: 151-168.

Peña, L.E., 1995. Revisión del género *Physogaster* Guérin, 1834 (Coleoptera: Tenebrionidae: Physogasterini). Gayana 59, 119‒130.

Pizarro-Araya, J., Jerez, V., 2004. Distribución geográfica del género *Gyriosomus* Guérin-Méneville, 1834 (Coleoptera: Tenebrionidae): una aproximación biogeográfica. Rev. Chil. Hist. Nat. 77, 491–500. http://dx.doi.org/10.4067/S0716-078X2004000300008

Platnick, N.I., Shadab, M., 1982. A revision of the American spiders of the genus *Camillina* (Araneae, Gnaphosidae). Am. Mus. Novit. 2748, 1‒38.

Platnick, N.I., Shadab, M.U., Sorkin, L.N., 2005. On the Chilean spiders of the family Prodidomidae (Araneae, Gnaphosoidea) with a revision of the genus *Moreno* Mello-Leitão. Am. Museum Novit. 3499, 1–31.

Ramírez, M.J., 2003. The spider subfamily Amaurobioidinae (Araneae, Anyphaenidae): A phylogenetic revision at the generic level. Bull. Am. Mus. Nat. Hist. 277, 1‒262. 10.1206/0003-0090(2003)277<0001:TSSAAA>2.0.CO;2

Roig-Juñent, S., Domínguez, M.C., 2001. Diversidad de la familia Carabidae (Coleoptera) en Chile. Rev. Chil. Hist. Nat. 74, 549‒571. http://dx.doi.org/10.4067/S0716-078X2001000300006.

Table S2 List of epigean arthropods from Paposo (Antofagasta Region, Chile).

Pizarro-Araya et al. 2021. A Fine-Scale Hotspot at the Edge: Epigean Arthropods from the Atacama Coast (Paposo-Taltal, Antofagasta Region, Chile).

Class	Orders	Families	Species	N
Arachnida	Acari	Indeterminate	Acari sp. 1	7
		Indeterminate	Acari sp. 2	1
		Indeterminate	Acari sp. 3	1
		Indeterminate	Acari sp. 4	1
		Indeterminate	Prostigmata sp.	2
		Indeterminate	Prostigmata sp. 1	2
		Caeculidae	*Andocaeculus* sp. 1	2
	Araneae	Indeterminate	Araneae sp. 1	1
		Araneidae	Araneidae sp. 1	1
		Araneidae	Araneidae sp. 2	1
		Araneidae	*Metepeira* sp. 1	4
		Thomisidae	Thomisidae sp.	1
		Gnaphosidae	Gnaphosidae sp. 1	4
		Gnaphosidae	Gnaphosidae sp. 2	1
		Sicariidae	*Loxosceles* sp. 1	1
		Sicariidae	*Sicarius* sp. 1	2
		Theridiidae	*Steatoda* sp. 1	3
		Anyphaenidae	*Amaurobioides* sp. nov.	1
	Pseudoscorpiones	Cheiridiidae	Cheiridiidae sp. nov.	1
	Scorpiones	Bothriuridae	*Bothriurus dumayi* (Cekalóvic, 1974)	3
			Brachistosternus barrigai (Ojanguren Affilastro & Pizarro-Araya, 2014)	1
			Brachistosternus paposo Ojanguren-Affilastro & Pizarro-Araya, 2014	8
			Brachistosternus roigalsinai Ojanguren-Affilastro, 2002	10
			Rumikirus sp. nov.	1
		Caraboctonidae	*Caraboctonus keyserlingi* (Pocock, 1893)	5
	Solifugae	Daesiidae	*Ammotrechelis* sp. 3 (sp. nov.)	1
		Mummuciidae	Mummuciidae sp. 1 (sp. nov.)	4
Insecta	Coleoptera	Anthicidae	Anthicidae sp. 1	35
		Buprestidae	*Atacamita chiliensis* (Laporte & Gory, 1835)	52

	Ectinogonia barrigai Moore 2017	1
Carabidae	*Calosoma* (*Castrida*) *vagans* Dejean, 1831	268
	Mimodromius sp. 1	4
	Mimodromius sp.1	10
	Mimodromius sp.2	1
Cerambycidae	*Neohebestola* sp. 1	1
Chrysomelidae	Chrysomelidae sp. 1	8
	Chrysomelidae sp. 1	1
	Lithraeus scutellaris (Philippi & Philippi, 1864)	2
Cleridae	*Inhumeroclerus thomsoni* Pic 1955	7
Corylophidae	Corylophidae sp. 1	3
Cryptophagidae	*Amydropa anophthalma* Reitter, 1877	3
	Amydropa sp. 1	1
Curculionidae	*Anthonomus* sp. 1	2
	Cnemecoelus sp. 1	4
	Curculionidae sp. 1	9
	Curculionidae sp. 1	1
	Curculionidae sp. 2	1
	Curculionidae sp. 2	1
	Curculionidae sp. 3	8
	Curculionidae sp. 4	2
	Curculionidae sp. 5	3
	Curculionidae sp. 6	1
	Curculionidae sp. 7	6
	Curculionidae sp. 8	1
	Curculionidae sp. 9	4
	Curculionidae sp. 10	1
	Curculionidae sp. 11	1
	Curculionidae sp. 12	1
	Cylydrorhinus sp. 1	1
	Hyperoides sp. 1	2

	Listroderes sp. 1	13
	Puranius sp. 1	1
	Scolytinae sp. 1	6
	Strangaliodes sp. 1	130
	Strangaliodes sp. 1	1
	Strangaliodes sp. 1	3
	Strangaliodes sp. 2	1
	Strangaliodes sp. 2	1
Elateridae	Elateridae sp. 1	1
Histeridae	*Halacritus riparius* (Bickhardt, 1914)	5
Latridiidae	*Dicastria temporalis* Dajoz, 1967	3
	Metophthalmoides castrii (Dajoz, 1967)	3
	Latridiidae n. sp. 1	1
	Latridiidae sp. 1	10
	Melanophthalma sp. 1	8
Leiodidae	Leiodidae sp. 1	101
Mauroniscidae	Mauroniscidae sp. 1	5
Meloidae	*Picnoseus* sp. 1	2
	Pseudomeloe chiliensis (Guérin-Meneville, 1830)	7
	Pseudomeloe sanguinolentus (Solier,1851)	7
Melyridae	*Arthrobrachus limbatus* Solier, 1849	1781
	Melyridae sp. 1	17
	Melyridae sp. 2	2
Mordellidae	*Mordellaria* sp. 1	5
Nitidulidae	Nitidulidae sp. 1	3
	Nitidulidae sp. 2	7
Oedemeridae	*Ananca cyanipennis* (Solier, 1851)	26
Phengodidae	*Mastinomorphus* sp. 1	27
Ptiliidae	*Mezium* sp. 1	1
	Ptiliidae sp. 1	1
	Ptiliidae sp. 3	1

	Smicrus sp.1	3
Ptinidae	Ptinidae sp. 1	1
	Xyletomerus sp. 1	1
	Xyletomerus sp. 1	2
Scarabaeidae	*Luispenaia paposo* Mondaca, Pizarro-Araya & Alfaro 2019	1
	Luispenaia sp. (sp. nov.)	1
Staphylinidae	*Omaliopsis* sp. 1	3
	Staphylinidae sp. 1	58
	Staphylininae sp. 1	1
Tenebrionidae	*Arthroconus* sp.	16
	Discopleurus baloghi Kaszab, 1969	3
	Entomochilus hirtipes (Kulzer, 1956)	29
	Geoborus lineatus Guérin-Méneville, 1834	2
	Gyriosomus angustus Philippi, 1864	79
	Gyriosomus curtisi Fairmaire, 1876	53
	Nycterinus (*Paranycterinus*) *angusticollis* Philippi,1864	5
	Nycterinus (*Paranycterinus*) *barriai* Peña,1971	2
	Nycterinus (*Paranycterinus*) *borealis* Peña,1971	29
	Nycterinus sp. 1	2
	Nycterinus sp. 3	2
	Physogaster globulus Solier, 1849	6
	Physogaster penai Kulzer, 1958	5
	Physogaster sp. 1	54
	Praocis (*Postpraocis*) sp.	2
	Psammetichus cekalovici Peña, 1973	38
	Psammetichus pilipes Guérin, 1834	4
	Scotobius brevipes Waterhouse, 1844	161
	Scotobius kaszabi Marcuzzi, 1976	6
	Scotobius sp. 1	25
	Scotobius tarapacensis Marcuzzi, 1976	9
	Thinobatis sp. 1	5

	Zopheridae	Zopheridae sp. 1	2
Collembola	Indeterminate	Collembola sp. 1	78
	Entomobryidae	Entomobryidae sp. 1	324
		Entomobryidae sp. 1	186
	Hypogastruridae	Hypogastruridae sp. 1	9
	Poduridae	Poduridae sp. 1	586
		Poduridae sp. 1	5
	Sminthuridae	Sminthuridae sp. 1	156
		Sminthuridae sp. 1	49
Hymenoptera	Bradynobaenidae	Bradynobaenidae sp. 1	2
		Bradynobaenidae sp. 2	2
	Formicidae	*Brachymyrmex* sp. 1	134
		Camponotus morosus (Smith, F., 1858)	14
		Camponotus sp. 1	6
		Dorymyrmex goetschi Goetsch, 1933	239
		Dorymyrmex pogonius (Snelling, 1975)	23
		Dorymyrmex sp. 1	1633
		Dorymyrmex sp. 1	105
		Formicidae sp. 1	2
		Formicidae sp. 1	37
		Formicidae sp. 1	343
		Formicidae sp. 2	112
		Formicidae sp. 2	1
		Solenopsis gayi Spinola, 1851	797
	Mutillidae	*Euspinolia militaris* Mickel, 1938	9
		Mutillidae sp. 1	10
		Mutillidae sp. 2	12
		Sphaerophtalma sp. 1	13
		Sphaerophtalma sp. 2	66
		Sphaeropthalma sp. 1	37
Orthoptera	Acrididae	*Trimerotropis ochraceipennis* (Blanchard, 1851)	1

	Mogoplistidae	*Hoplosphyrum griseus* (Philippi, 1863)	137
		Microgryllus pallipes (Philippi, 1863)	51
	Ommexechidae	*Conometopus penai* Ronderos, 1972	4
		Conometopus sp. 1	5
	Proscopiidae	*Astromoides* sp. 1	1
	Tettigoniidae	*Anysophia* sp. 1	11
		Phaneropterinae sp. 1	1
		Platydecticus sp. 1	3
		Platydecticus sp. 1	1
		Platydecticus sp. 1	19
	Tristiridae	*Elasmoderus lutescens* (Blanchard, 1851)	19
		Enodisomacris curtipennis Cigliano, 1989	1
Psocoptera	Liposcelidae	Liposcelidae sp. 1	1
		Liposcelidae sp. 1	4
	Indeterminate	Psocoptera sp. 1	116
	Indeterminate	Psocoptera sp. 1	1
	Indeterminate	Psocoptera sp. 1	350
	Indeterminate	Psocoptera sp. 2	68
Thysanura	Lepismatidae	Thysanura sp. 1	43
		Lepismatidae sp. 1	13
